# The *Aspergilli* and Their Mycotoxins: Metabolic Interactions With Plants and the Soil Biota

**DOI:** 10.3389/fmicb.2019.02921

**Published:** 2020-02-12

**Authors:** Walter P. Pfliegler, István Pócsi, Zoltán Győri, Tünde Pusztahelyi

**Affiliations:** ^1^Department of Molecular Biotechnology and Microbiology, Institute of Biotechnology, Faculty of Science and Technology, University of Debrecen, Debrecen, Hungary; ^2^Institute of Nutrition, Faculty of Agricultural and Food Sciences and Environmental Management, University of Debrecen, Debrecen, Hungary; ^3^Central Laboratory of Agricultural and Food Products, Faculty of Agricultural and Food Sciences and Environmental Management, University of Debrecen, Debrecen, Hungary

**Keywords:** *Aspergillus*, aflatoxin, mycotoxin, plant, insect, microbe, soil, interaction

## Abstract

Species of the highly diverse fungal genus *Aspergillus* are well-known agricultural pests, and, most importantly, producers of various mycotoxins threatening food safety worldwide. Mycotoxins are studied predominantly from the perspectives of human and livestock health. Meanwhile, their roles are far less known in nature. However, to understand the factors behind mycotoxin production, the roles of the toxins of *Aspergilli* must be understood from a complex ecological perspective, taking mold-plant, mold-microbe, and mold-animal interactions into account. The *Aspergilli* may switch between saprophytic and pathogenic lifestyles, and the production of secondary metabolites, such as mycotoxins, may vary according to these fungal ways of life. Recent studies highlighted the complex ecological network of soil microbiotas determining the niches that *Aspergilli* can fill in. Interactions with the soil microbiota and soil macro-organisms determine the role of secondary metabolite production to a great extent. While, upon infection of plants, metabolic communication including fungal secondary metabolites like aflatoxins, gliotoxin, patulin, cyclopiazonic acid, and ochratoxin, influences the fate of both the invader and the host. In this review, the role of mycotoxin producing *Aspergillus* species and their interactions in the ecosystem are discussed. We intend to highlight the complexity of the roles of the main toxic secondary metabolites as well as their fate in natural environments and agriculture, a field that still has important knowledge gaps.

## Introduction

The lifestyles of *Aspergillus* species associated with plants range from saprophytes and symptomless endophytes to weak and opportunistic phytopathogens. The shift between these lifestyles is the result of global transcriptome changes, primarily affecting secondary metabolite (SM) production (e.g., Reverberi et al., 2013). The principal and well-known mycotoxins produced by the *Aspergilli* are ochratoxin A (OTA) and aflatoxins (AFs), as well as less-prominent toxins like patulin (Keller et al., 2005). These toxins are found in different agricultural commodities (Varga et al., 2004), and are tightly regulated with different threshold limits depending on the matrix (Cano et al., 2016).

Due to the importance of SMs in plant pathogenesis and animal toxicoses, understanding their regulation and biosynthesis is crucial but still hindered by notable knowledge gaps. The species *A. flavus*, for example, has been predicted to possess 56 SM biosynthesis gene clusters (Keller et al., 2005), but only some secondary metabolites, e.g., AFs (Yu et al., 2004), aflatrem (Nicholson et al., 2009), piperazine (Forseth et al., 2013), asparasone (Malysheva et al., 2014), cyclopiazonic acid (CPA) (Chang et al., 2009), and kojic acid (Terabayashi et al., 2010) have been assigned to a particular gene cluster (Ehrlich and Mack, 2014). *A. flavus* thus might produce metabolites besides well-known mycotoxins that could be underrated contributors to its toxicity to humans and animals.

Initially, it was hypothesized that mycotoxin production helps fungi to compete with other organisms for nutrient sources like fruits or seeds (Janzen, 1977). Mycotoxins are now also known to act as chemical signals between representatives of different kingdoms, e.g., as inhibitors of quorum sensing (QS), virulence factors in pathogens, or as protectors of sclerotia from insect predation (Ciegler, 1983; Wicklow et al., 1994; Desjardins and Hohn, 1997; Rasmussen et al., 2005; Rohlfs et al., 2010).

Due to their economic and public health importance, the research on mycotoxins has so far mostly been focused on animal husbandry, the food chain, and human aspects. However, for a comprehensive understanding of toxigenic molds’ ecology and of the evolutionary pressures shaping mycotoxin production, interactions with the micro- and macroflora and fauna in different habitats need to be considered and investigated. The study of the overall role of microbial SMs in natural habitats is a previously mostly neglected, but an emerging field (O’Brien and Wright, 2011).

## *Aspergillus* Mycotoxins and Their Ecological Roles

### Sterigmatocystin/Aflatoxins

AFs are produced by as much as 16 species (Frisvad et al., 2019), most notably by *A. flavus* and *A. parasiticus*. A wide range of *Aspergillus* spp. produces the AF precursor sterigmatocystin (ST), which is also a carcinogenic compound. The ST/AF polyketide biosynthetic pathways are perhaps the most thoroughly studied ones in fungi (Cleveland et al., 2009; Khaldi et al., 2010).

The most common AF-producing species and the most common member of section *Flavi* is *A. flavus*, which possesses two distinct morphotypes, namely the “L-type” with big sclerotia (with average diameter of >400 μm), and the “S-type” that produces small sclerotia (under 400 μm) (Gilbert et al., 2018). However, several additional and often newly delimited species (*A. aflatoxiformans, A. arachidicola, A. austwickii*, *A. cerealis, A. minisclerotigenes, A. mottae, A. pipericola,* and *A. texensis*) have been characterized by S-type sclerotia. Earlier reports on S-type *A. flavus* may have referred to any of these species, including those that produce both aflatoxin B1 (AFB1) and aflatoxin G1 (AFG1) (so-called SBG strains) (Singh et al., 2018; Frisvad et al., 2019).

While the ecological role of ST is not known in detail, it is presumably antagonistic to organisms competing for resources with ST producers. Both AFs and ST have been reported to be phytotoxic (Stoessl, 1981; McLean et al., 1995). AFs inhibit plant photosynthesis by hindering chlorophyll and carotenoid synthesis (Anjorin and Inje, 2014), leading to virescence or albinism in the contaminated plants (Reiss, 1978). However, in plant pathogenesis, the role of these mycotoxins needs to be investigated as non-aflatoxigenic strains also have the potential to colonize plant hosts, e.g., on cotton bolls (Cotty, 2007), and these types of strains are isolated frequently.

Soil is the natural habitat for *A. flavus*, and AF production is considered to give a fitness advantage in that environment (Drott et al., 2017). Selective forces that maintain the polymorphism of non-aflatoxigenic and aflatoxigenic colonies are mainly unknown. Resource competition among the closely related strains is modulated by factors such as chemical composition and pH of the soil or nutrient and water availability (Ehrlich, 2014). Moreover, competition between aflatoxigenic and non-aflatoxigenic strains is strain-dependent, and it must be noted that non-aflatoxigenic strains are not necessarily atoxigenic, as they may produce toxins other than AFs. Under high fungal density, non-aflatoxigenic strains can outcompete both toxigenic and other non-aflatoxigenic populations (Cotty, 2006). Aflatoxigenic isolates were shown to have lower fitness than non-aflatoxigenic isolates in wide temperature ranges (25–42°C) (Drott et al., 2019). This may explain the success of the latter in competition. The metabolic cost of AF production seems to explain the low fitness as AFB1 itself does not affect the growth of *A. flavus* at concentrations as high as 500 ng g^−1^ (Drott et al., 2019), orders of magnitude higher than what can be measured in soils (0.6–5.5 ng·g^−1^) (Accinelli et al., 2008). Inoculation of soil with non-aflatoxigenic strains also protects crops from AF contamination during storage (Dorner and Cole, 2002; Bandyopadhyay et al., 2016).

AFB1 is transient in soils with a half-life of approximately 5 days at 28°C; however, it is produced continuously as long as there is a substrate, e.g., corn residues (Accinelli et al., 2008). High *A. flavus* levels (log10 3.1–4.5 cfu·g^−1^), AFB1 production, and expression of the AF biosynthetic genes (*aflG, aflD, aflP, aflR*, and *aflS*; Ehrlich et al., 2005) have been reported in the former study.

**Figure 1:**
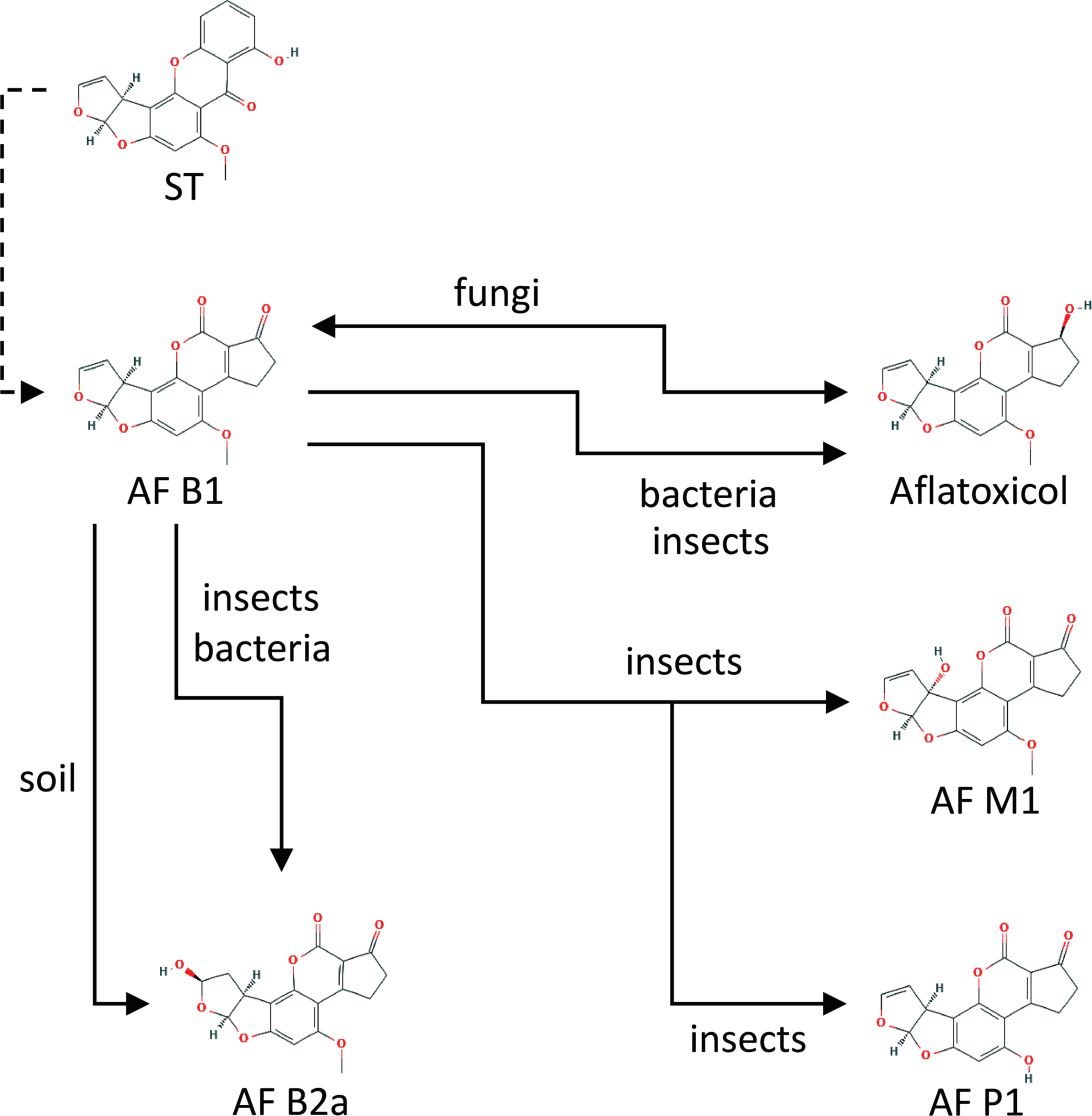
Main chemical conversions of aflatoxin B1 (AFB1) under interaction with different organisms and soil. Sterigmatocystin (ST) is a chemical precursor of aflatoxin B1 (AFB1) in aflatoxigenic fungi. The further conversion processes are explained in details in the text. Source: National Center for Biotechnology Information. PubChem Compound Database (accessed June 6, 2019) (Bolton et al., 2008).

Studies on AFB1 transformation in soil or purified mineral systems have identified AFs B2 (AFB2) and G2 (AFG2) as the primary transformation products using thin-layer chromatography. However, the more sophisticated HPLC-MS technique did not detect these molecules in spiked soils. In an aqueous-soil environment, a new structure, B2a (AFB2a), was detected as the single primary transformation product. AFB2a is a hydrolytic product of AFB1 and the soil acting as an acid catalyst (Starr et al., 2017) (Figure 1).

AFs taken up through plant roots can be accumulated, transported to other tissues (e.g., in groundnut seedlings; Hariprasad et al., 2015; Snigdha et al., 2015), degraded, metabolized, or masked, or can be diffused back to the medium (e.g., in maize; Mertz et al., 1980).

Various fungi can inhibit AF accumulation. In an *in vitro* soil environment, *Fusarium oxysporum* was able to inhibit AF production at different temperatures (25 and 30°C) and fumonisins accumulated instead of AFB1 (Falade et al., 2016). On the contrary, inhibitory effect by *A. flavus* on *Fusarium oxysporum* f. sp. *niveum* and *Fusarium solani* f. sp. *cucurbitae* has also been described with an inhibition rate exceeding 50 % in *in vitro* and greenhouse experiments. Hyperparasitism of *A. niger, A. flavus*, and *A. terreus* on *F. oxysporum* f. sp. *melonis* was also demonstrated (Boughalleb-M’Hamdi et al., 2018).

### Gliotoxin

Gliotoxin (an epipolythiodioxopiperazine) has internal disulfide bridges that conjugate proteins (Spikes et al., 2008). Gliotoxin biosynthesis and regulation are reviewed by Dolan et al. (2015). The compound is implicated in the formation of reactive oxygen species (ROS) by redox cycling and is generally broadly cytotoxic (Gardiner et al., 2005). Therefore, its detoxification is only possible by its biosynthetic enzymes (Scharf et al., 2018). One of the significant gliotoxin producers besides biocontrol *Trichoderma* ssp. is *A. fumigatus*, a saprophyte and an opportunistic animal pathogen. Gliotoxin produced by this fungus acts as a virulence factor mediating systemic mycosis in susceptible vertebrates (Latgé, 2001; Scharf et al., 2016) and presumably in insects (Reeves et al., 2004). *A. fumigatus* possesses a self-protecting system against gliotoxin (Schrettl et al., 2010; O’Keeffe et al., 2014). RNA-seq revealed 164 differentially expressed genes (DEGs) in *A. fumigatus* treated with external gliotoxin, and besides gliotoxin biosynthesis genes, helvolic acid biosynthesis genes, siderophore-iron transport genes showed altered expression (O’Keeffe et al., 2014). High temperature and humidity during crop maturation may favor *A. fumigatus* presence and toxin production. Gliotoxin enters the food chain and reaches the most sensitive farm animals, like horses and poultry (Pena et al., 2010). However, there is no threshold limit for this molecule.

In composted mineral soil with a natural microbiota, the toxin may function as an antibiotic, effectively controlling the damping-off disease of *Zinnia elegans* (zinnia) seedlings caused by the fungus *Rhizoctonia solani* and the water mold *Pythium ultimum* (Lumsden et al., 1992). A strong correlation between the presence of bacterial peptidoglycan, lipopolysaccharide, or lipoteichoic acid in soil and the gliotoxin secretion of *A. fumigatus* was described by Svahn et al. (2014). This finding was potentially relevant for drug discovery research, and parallelism was found with the increased virulence of *A. fumigatus* in case of bacterial co-infection.

### Ochratoxins

Several *Aspergilli* in sections *Circumdati* (such as *A. steynii* and *A. westerdijkiae*), *Flavi*, and *Nigri* (e.g., *A. carbonarius* and *A. niger*; Palencia et al., 2010) are well-known producers of OTA, a mycotoxin teratogenic, carcinogenic, immunosuppressive, and nephrotoxic in animals (Samson et al., 2014). All studied OTA-producing fungi have a consensus OTA biosynthetic pathway with four highly conserved biosynthetic genes in a cluster and a bZIP transcription factor (Wang et al., 2018).

OTA induced necrotic lesions on *Arabidopsis thaliana* leaves *via* induction of an oxidative burst by elevated ROS (hydrogen peroxide and superoxide anion) levels (Peng et al., 2010). Meanwhile, the downregulation of the antioxidant defense enzymes in host plants and up-regulation of lipid peroxidation were detected, along with root growth inhibition of seedlings (Peng et al., 2010). Infiltration of 4-week-old *A. thaliana* leaves with 2 mM and 1 mM OTA solutions *in vitro* resulted in macroscopic lesions (Wang et al., 2012), and the growth of *A. thaliana* was repressed, while cell death was detected with characteristic hypersensitive response-type lesions on the excised leaves. Cell death did not only result in a manifestation of oxidative burst but the deposition of phenols and callose (Peng et al., 2010) as well. McLean (1996) investigated the effect of the toxin on germinating *Zea mays* embryos. Interestingly, there was no linear relationship between the inhibitory effect and the OTA concentrations as 10 μg·ml^−1^ OTA was inhibitory, while 5 or 25 μg·ml^−1^ OTA was stimulatory for root and shoot growth.

Soil type, in connection with microbial activity, affects OTA half-life. In soils with higher microbial activity, like planted soils, faster degradation could be measured (Mortensen et al., 2006) caused by the microbial biomass (e.g., Barberis et al., 2014). Regulation of OTA biosynthesis can be modulated by volatile organic carbons (VOCs) as observed for *A. carbonarius* and fruit ketones, C-8 alcohols, and trans-nerolidol (Zhang et al., 2017).

### Patulin

Patulin is a polyketide mycotoxin produced by *Penicillium* spp. and to a lesser extent, various *Aspergilli* (Zhang et al., 2008). It is frequently found in fresh fruits or fruit juices and jams contaminated with blue mold rot (Logrieco et al., 2003). Like clavatol, patulin inhibits numerous plant pathogenic fungi and water molds *in vitro*, i.e., *Fusarium oxysporum* f. sp. *cucumerinum*, *Botrytis cinerea, Didymella bryoniae, Rhizoctonia solani*, and *Pythium ultimum* (Zhang et al., 2008). Patulin and clavatol produced by *Aspergillus clavatonanicus* endophyte of *Taxus mairei* have been shown to antagonize plant pathogens (Zhang et al., 2008). Interestingly, Botha et al. (2018) reported that *A. clavatus* produced higher concentration of tremorgenic mycotoxins (i.e., tryptoquivaline A, deoxytryptoquivaline A, and deoxynortryptoquivaline) than concomitant patulin and cytochalasin E. Patulin, similarly to penicillic acid has the potential to interfere with bacterial QS communication in soil (Rasmussen et al., 2005), hinting at its potentially manifold ecological roles in microbial communities.

### Cyclopiazonic Acid

The neurotoxic CPA is an indole-tetramic acid produced by 13 species in section *Flavi* (Frisvad et al., 2019). It inhibits endoplasmic reticulum calcium ATPases at nanomolar concentrations, and therefore, it is an inducer of cell death in plants (Chang et al., 2009). Usually, CPA and AFs are concomitant mycotoxins. Most *A. flavus* strains synthesize AFs B1 and B2 besides CPA, although some strains also synthesize AFs G1 and G2 (Geiser et al., 2000; Cardwell and Cotty, 2002). In contrast, *A. parasiticus* strains produce all four AFs without CPA biosynthesis (Dorner et al., 1984). Moreover, a “sleeping” CPA cluster was activated by the overexpression of a general secondary metabolism regulator gene (*laeA*) in *A. fumisynnematus* (Hong et al., 2015).

CPA was proposed to modify calcium homeostasis, mitochondria, and cytoplasm membranes based on animal studies (Riley and Goeger, 1992). This mycotoxin serves as a critical pathogenicity factor that enables the saprophytic lifestyle of *A. flavus* (Chalivendra et al., 2017), presumably, through its good iron-chelating characteristics (Riley and Goeger, 1992).

## Plant-Fungal Interactions

### Peanut-*Aspergillus flavus* Interaction

It is well-known that multiple mechanisms are involved in host plant defense systems in response to *A. flavus* infection and AF accumulation. Peanut was found to have evolved complex defense mechanisms to resist pathogens, such as blocking the invasion and activating a range of defense responses (Holbrook and Stalker, 2003). Eight hundred forty-two candidate genes were recognized for *A. flavus* resistance in post-harvest seeds (Wang et al., 2016a). Genes involved in defensive responses to *A. flavus* and AF biosynthesis were stimulated in resistant genotype (Wang et al., 2016b).

The plant cell wall, the first line of defense against microbial pathogens, is primarily made up of polysaccharides cellulose, hemicellulose, and pectin. While opportunistic fungi usually infect plants through wounds (e.g., mechanical or pest damages), pathogenic ones actively penetrate cell walls, often through the secretion of a range of polysaccharide-degrading enzymes such as pectinesterase, arabinofuranosidase, mannosidase, and galacturonidase along with amylases or proteases (Whitehead et al., 1995; Bellincampi et al., 2014; Wang et al., 2016b). In peanuts resistant to *A. flavus* infection, feruloyl esterase, pectinesterase, arabinofuranosidase, mannosidase, polygalacturonase, and galacturonidase fungal activities were significantly downregulated compared to the sensitive plants (Wang et al., 2016a). Resistance to *A. flavus* infection is naturally the most critical factor in avoiding AF exposure to consumers. Pod infection, seed invasion, and AF production in the cotyledon are the crucial steps to be considered (Nigam et al., 2009). The first interaction between the plant and the mold is at the pod shell, where the pathogen resistance depends on the shell structure. The second barrier is the undamaged seed coat. Upon a successful invasion, *A. flavus* colonizes the seed cotyledon and produces AFs. In a proteomic study, a total of 29 seed proteins showed differential expression between the resistant and susceptible peanut cultivars under drought stress in response to *A. flavus* (Wang et al., 2010). Under drought stress, AF production was consistent in peanut pods even if roots of those plants were well watered. Meanwhile, AF was not produced in well-watered peanuts pods, while roots were under drought stress (Sanders et al., 1993).

The data suggest that drought stress is the most critical factor in the interaction of the plant and the fungal agent. Therefore, watering of the fields is crucial along with the improvement of the plant’s resistance by genetic modification or selection.

### Maize-*Aspergillus flavus* Interaction

Pathogenesis in maize depends on environmental factors (e.g., Payne and Widstrom, 1992; Kebede et al., 2012; Fountain et al., 2014), metabolic state of the kernels (Chen et al., 2010; Jiang et al., 2011), physiological state of the fungus (Jayashree and Subramanyam, 2000), and time elapsed following infection (Scott and Zummo, 1994; Betrán and Isakeit, 2004). Vitreous compared to softer dent type endosperm was positively correlated with AF contamination and resistance to ear rot (Betrán and Isakeit, 2004; Llorente et al., 2004).

Since maize is a favorable host for the *Aspergilli*, especially for *A. flavus*, and the plant’s resistance is genetically determined, much effort was invested worldwide to develop resistant maize genotypes. Recent breeding investigations focused on quantitative trait loci (QTL) for AF resistance (Kelley et al., 2012; Fountain et al., 2015), and the studies demonstrated that the resistance to *A. flavus* is highly quantitative and is not conferred by a single gene. Any given QTL was found to account for a rather low level of phenotypic variance explained regarding AF resistance. Resistance thus has a polygenic nature with a combination of multiple traits being involved in the resistant phenotype (Fountain et al., 2014; Yin et al., 2014). Maize inbred lines were found also to vary in their tolerance to CPA (Chalivendra et al., 2017). Moreover, CPA tolerance of the root was in a significant correlation to silk resistance under fungal colonization (Mideros et al., 2012).

During infection, mycelia were detected inside the scutellum, exhibiting a biofilm-like formation at the endosperm-scutellum interface (Dolezal et al., 2013). This biofilm-like structure bears resemblance to the biofilm of *A. fumigatus* in the human lung (Loussert et al., 2010). *In situ* hybridization of RNA showed the expression of the pathogenesis-related protein gene in the aleurone and scutellum of maize seed (PRms) during *A. flavus* infection (Shu et al., 2015). Transcripts of the maize sucrose synthase-encoding gene (shrunken-1; Sh1) were detected in the embryo in non-infected kernels, but the gene was up-regulated in the aleurone and scutellum under *A. flavus* infection. Moreover, the transcripts of PRms and Sh1 showed accumulation in the seeds before infection (Shu et al., 2015).

A recent study was conducted on expression profiling of 267 unigenes (mostly genes of metabolism, stress response and disease resistance) in a mapping population derived from a cross between susceptible and resistant parent plants (Dhakal et al., 2017). It revealed that many genes involved in the synthesis and hydrolysis of starch and sugar mobilization and others related to energy production and/or precursors of lignin and phytoalexins used in the defense response were highly expressed (Dolezal et al., 2014; Shu et al., 2015; Dhakal et al., 2017).

Apart from *Fusarium* infection (Mesterházy, 2008), *A. flavus* causes the most economic loss on cornfields. However, co-infection by these genera is not investigated in detail, and only some aspects are known like the inhibitory effect on AFB1 production by *Fusarium* (Falade et al., 2016), and inhibitory and hyper-parasitic effect of *A. flavus* on Fusaria (Boughalleb-M’Hamdi et al., 2018). Moreover, the physiological effects of the co-produced mycotoxins like CPA and AFs or the effect of the co-infection on mycotoxin productions is rarely investigated (e.g., Marín et al., 2001; Giorni et al., 2016).

### Cotton-*Aspergillus flavus* Interaction

Cottonseed can be contaminated pre-and postharvest by *Aspergilli*. A comparative transcriptome analysis was performed investigating the genes expressed differentially in corn, peanut, and cotton under aflatoxigenic *A. flavus* infection (Bedre et al., 2015). Only 26 common genes were identified as candidate *A. flavus* resistance genes in all the three plants. Six of these genes coded for Fe(II)-dependent oxygenase superfamily proteins and 2-oxoglutarate. In response to both non-aflatoxigenic and aflatoxigenic strains, genes encoding alcohol dehydrogenase, UDP glycosylation transferase, and helix loop helix protein were induced (Bedre et al., 2015). Upregulation of primary metabolism modulated signal transduction cascades that were essential to plant defense responses (Rojas et al., 2014). In the pericarp, sucrose and starch metabolism besides glycerolipid metabolism were upregulated under infection with non-aflatoxigenic *A. flavus*. The metabolic pathways activated by the presence of non-aflatoxigenic *A. flavus* in the plant pericarp and seeds compared to aflatoxigenic *A. flavus* activated pathways can lead to possible target genes to develop fungal stress tolerance and resistance in cotton (Bedre et al., 2015).

### Phytohormone Guided Interactions

Phytohormones are well-known mediators of fungus-plant interactions with different roles. The abscisic acid (ABA) (Hauser et al., 2011; Xin et al., 2012), salicylic acid (SA) (Janda and Ruelland, 2014), and ethylene (ET) (Bleecker and Kende, 2002; Ton et al., 2002) phytohormonal pathways in plants can act against *A. flavus* and AF production by mediating and channeling many stress-response genes (Bari and Jones, 2009). Transcriptomic analysis revealed DEGs of phytohormone production and signaling in response to AF production in peanut (Wang et al., 2016a). Moreover, DEGs concerning ABA production and signaling showed higher expression in a sensitive peanut genotype than in the resistant plants (Wang et al., 2016b).

Determining the roles of ET is challenging as disease symptoms seem to be either reduced or enhanced or not affected depending on the pathogen-host interaction (Bleecker and Kende, 2002). It inhibits AF biosynthesis in *A. flavus* through alleviation of oxidative stress (Huang et al., 2009). However, DEGs involved in ET production were downregulated in response to AF production, and most of them were also repressed in the resistant genotype. Wang et al. (2016b) concluded that ET might suppress resistance to AF production, and later Wang et al. (2017) found that ET emitted by infected seed facilitated the colonization by *A. flavus* but not AF production in maize, potentially opening up biotechnological applications.

Contrary, SA is suppressive for some fungi (Seyfferth and Tsuda, 2014). SA inhibited mycelial growth and mycotoxin formation of *A. flavus in vitro*, and the *in vivo* evaluation resulted in more significant inhibitory effects for the intact treated pistachio fruit as for injured ones (Panahirad et al., 2014).

Jasmonates are lipid-derived signals compounds in plant growth and development in response to stresses like pathogen attack or drought (Wasternack, 2014). Jasmonic acid (JA) and its metabolites, members of the oxylipin family, are synthesized in the alpha-linolenic acid pathway. Many of them modify gene expression in a regulatory network with synergistic and antagonistic effects concerning other plant hormones such as SA, auxin, ET, and ABA (Wasternack, 2007). Metabolism of alpha-linolenic acid was upregulated in pericarp under both non-aflatoxigenic and toxigenic *A. flavus* infection in comparison to seeds. Similarly, the alkaloid biosynthetic pathway was more intensively upregulated in the pericarp under both non-aflatoxigenic and toxigenic *A. flavus* infection than in the seed. In tobacco host plants, the alkaloid biosynthesis was increased in response to insect foraging and application of JA (Todd et al., 2010). Therefore, it was suggested that the JA-regulated defense response is also stimulated as an answer to *A. flavus* infection (Bedre et al., 2015).

Furthermore, in the case of the aflatoxigenic *A. flavus* infection, upregulation of arachidonic acid (AA) metabolism was detected in seeds, exceeding that under non-aflatoxigenic infection in the pericarp. AA has a role in plants as a signaling compound, and it stimulates plant defense responses through fatty acids. Meanwhile, pathogen AA triggers plant innate immunity resulting in defense responses and programmed plant cell death (Savchenko et al., 2010).

### Pathogenesis-Related (PR) Proteins

PR proteins are disease resistance proteins induced in the host plant in response to pathogen infection (Bravo et al., 2003; Luo et al., 2011). Identification and characterization of such plant genes have importance in reducing fungal pathogenicity. In maize, PR-protein genes included PR-1, PR-4, PR-5, PR-10, and chitinase (Dhakal et al., 2017).

The plant hydrolytic enzymes like *β*-1,3-glucanases and chitinases show antifungal activity owing to the degradation of fungal cell wall components (Cordero et al., 1994; Dolezal et al., 2014). Plant chitinases also have lysozyme activity and are active in preventing mycelial development (Collinge and Slusarenko, 1987; Collinge et al., 1993). The gene expression of chitinase 2 and PR-10 was reported to be upregulated in maize seeds during fungal infection (Cordero et al., 1994). *In vitro* PR-10 protein possessed antifungal activity against *A. flavus*, and its production was upregulated upon *A. flavus* infection in a resistant maize hybrid but not in a susceptible one (Chen et al., 2006). RNAi gene silencing driven repression of PR-10 resulted in an increased susceptibility to *A. flavus* and AF production (Chen et al., 2010). Moreover, overexpression of chitinase genes (Cletus et al., 2013) resulted in resistance against fungal infection in rice (Baisakh et al., 2001) and peanut (Rohini and Sankara Rao, 2001; Prasad et al., 2013).

Besides chitinases (Singh et al., 2015), lectins are also involved in the plant defense mechanisms (Dang and Van Damme, 2015) and probably play an essential role in inhibiting AF production (Hawkins et al., 2015). In resistant and sensitive plant genotypes, chitinase showed different expression levels (Wang et al., 2016a). Eleven chitinase encoding transcripts were expressed differentially in pericarp and seed during infection by both aflatoxigenic and non-aflatoxigenic strains in cotton (Bedre et al., 2015), while in maize seven chitinase genes were associated with the increased *in vivo* resistance to *A. flavus* infection and AF accumulation (Hawkins et al., 2015).

Production of the PR maize seed protein, ZmPRms, was recently shown to be involved in resistance to *A. flavus* and other pathogens in a seed-specific RNA interference study (Majumdar et al., 2017). *A. flavus* infection increased significantly on corn kernels with downregulated *ZmPRms* with a concomitant 4.5–7.5-fold higher accumulation of AFs, presenting the protein’s role in evading infection and toxin accumulation (Majumdar et al., 2017).

Plants also produce cell wall polygalacturonase-inhibiting proteins to counteract the activity of fungal polygalacturonases (Kalunke et al., 2015), enzymes that catalyze the hydrolysis of the *α*-(1–4) linkages between the D-galacturonic acid units in homogalacturonan resulting in cell separation in the plant tissues. The interaction between polygalacturonases and inhibiting proteins promoted the formation of oligogalacturonides, which evoked further defense responses (Federici et al., 2006). In peanut, Wang et al. (2016b) showed that all six DEGs of polygalacturonase-inhibiting proteins were upregulated to a much higher level in a resistant genotype than in a sensitive one.

### Oxylipins

Plant’s linoleic acid and 9- and 13-hydroperoxy fatty acids (9S- and 13S-HPODE oxylipin products) have a substantial effect on the differentiation processes of *Aspergillus* spp. Both 9S- and 13S-HPODE alter secondary metabolism in *A. parasiticus* and *A. nidulans* (Gardner, 1995; Burow et al., 1997). They also increase the production of the conidiospores in *A. nidulans* and *A. flavus*, and, in *A. nidulans*, elevate cAMP levels (Calvo et al., 1999; Affeldt et al., 2012). Additionally, *A. flavus* infection of peanut seeds promoted linoleate 9-LOX expression and 9S-HPODE accumulation. 13S-HPODE producing lipoxygenase alleles (PnLOX2 and PnLOX3) were highly expressed in mature seed, but these genes were repressed between 5-fold and 250-fold during *A. flavus* infection. The outcomes of these investigations proposed that 9S-HPODE is a susceptibility, while 13S-HPODE is a resistance factor during *Aspergillus* spp. infection (Tsitsigiannis et al., 2005). Similarly, linoleic acid host-derived oxylipins were also suggested to drive mycotoxin synthesis (Burow et al., 1997; Brodhagen et al., 2008; Reverberi et al., 2010). 13S-HPODE repressed expression of ST and AF biosynthetic pathway genes at concentrations of 10 and 100 μM and, in this way, significantly reduced ST and AF production in both *A. nidulans* (ST producer) and *A. parasiticus* (AF producer) *in vitro* (Burow et al., 1997). The maize ZmLOX3-mediated pathway acted as a root-specific suppressor of all three major defense signaling pathways (Gao et al., 2008a,b).

The oxylipin-driven processes are complicated further by fungal oxylipin production. *A. flavus* single lipoxygenase produced oxylipins influence host responses. Reverberi et al. (2010) found that a lox-like gene mutant *A. ochraceus* was not only failed to produce 13S-HPODE, but a sharp decrease was detected in its OTA production. The conidium formation was also delayed, and the sclerotium production was increased in the cultures. Moreover, seeds infected with the *A. ochraceus* mutant could not produce normal 9S-HPODE levels or induce the defensive PR1, suggesting the importance of the fungal 13S-HPODE in the regulation of host defense response. The oxylipin profile of the maize kernels inoculated with wild type and *lox* mutant *A. flavus* strains showed elevated levels of HPODE and diHODES, also suggesting that the fungal Lox produces compounds that suppress plant oxylipin production. The *ΔAflox1* mutant strain was able to produce AF only on kernels, but not in axenic culture (Scarpari et al., 2014), revealing the complexity of the metabolic interactions.

PSIB *α* oxylipins derived from linoleic acid in *A. nidulans* were also reminiscent of those produced from seed fatty acids, and the infected seeds were able to influence the fungal development imitating and interfering with signals controlling conidiogenesis (Prost et al., 2005).

### Antioxidants

Oxidative stress is a critical factor that can stimulate the synthesis of AF and other SMs (Reverberi et al., 2010, 2013). H_2_O_2_ and other oxidative agents (Fanelli et al., 1985; Jayashree and Subramanyam, 2000; Narasaiah et al., 2006) activate AF biosynthesis in *Aspergillus* sect. *Flavi* (Reverberi et al., 2008). At the plant-pathogen boundary, ROS production is an essential feature that contributed to *Aspergillus* virulence besides SM production (Reverberi et al., 2013). In seeds contaminated with Aspergilli, a burst of H_2_O_2_ was detectable within a few hours of infection (Lamb and Dixon, 1997; Kachroo et al., 2003; Reverberi et al., 2008; Peng et al., 2010). For *A. flavus*, it appeared that lowering H_2_O_2_ levels in the corn embryo helps to prevent *A. flavus* infection and AF accumulation (Magbanua et al., 2007).

Among the stress-related transcripts, the presence of fungal superoxide dismutase in the dent samples indicated oxidative stress, known to be coupled to the production of AFs (Jayashree and Subramanyam, 2000; Fountain et al., 2015, 2016). It is arising that oxidative stress in fungi plays an essential role not only in SM biosynthesis but also in plant-fungal interactions. Within plant tissues, environmental stresses, e.g., drought and heat stress, may also result in the accumulation of ROS and play an essential role in communication between plants and the Aspergilli (Fountain et al., 2014).

In various plant seeds (e.g., maize, sunflower), the processes of lipoperoxidation induce a change in the ratio of oxidants and antioxidants, in favor of ROS accumulation in fungal cells and stimulating synthesis of AFs in *A. flavus* and *A. parasiticus* (Fabbri et al., 1983; Burow et al., 1997; Reverberi et al., 2008; Gao and Kolomiets, 2009). The SM production may be considered as the result of fungal cell response to incomplete scavenging of ROS (Reverberi et al., 2008; Hong et al., 2013).

At the plant’s side, DEGs and antioxidant transcripts of glutathione S-transferase, ferredoxin, copper amine oxidase, ascorbate peroxidase, and peroxidase involved in ROS processing and scavenging showed amplified activity during infection with both non-aflatoxigenic and toxigenic *A. flavus* (Bedre et al., 2015). Plant peroxidases also contributed to the response to AF production. DEG peroxidases showed a significantly higher expression in an *A. flavus* resistant peanut genotype than in a sensitive one, indicating better management of ROS in the former during fungal infection (Wang et al., 2016a).

Genes of the phenylpropanoid biosynthetic pathway that produce antimicrobial phytoalexins, phenolic substances, and lignin in plants (Collinge and Slusarenko, 1987; Lawton and Lamb, 1987) were found to show higher expression and more rapid activation in an *A. flavus* resistant maize genotype than in a sensitive one. Moreover, biosynthesis genes of phenylpropanoids, flavonoids, stilbenoids, diarylheptanoids, and gingerol were enriched only in the resistant maize genotype (Wang et al., 2016a). DEGs analysis in cotton inoculated with aflatoxigenic and non-aflatoxigenic *A. flavus* also revealed some significant variances in the expression rates of the genes taking part in the defense mechanisms. For instance, in the pericarp, the phenylpropanoid pathway was enriched at a higher level under aflatoxigenic strain infection than under non-aflatoxigenic infection (Bedre et al., 2015).

The flavonoid pathway is essential in the production of several antifungal compounds and, therefore, it is related to defense reactions (Treutter, 2005). In seeds, the flavonoid biosynthesis pathway was the utmost upregulated under non-aflatoxigenic *A. flavus* infection exceeding the pericarp (Bedre et al., 2015). Numerous studies illustrated the potential impact that flavonoids could exert on SM production. Rutin (quercetin-3-rutinoside) was demonstrated as an effective inhibitor of AFB1 production (Chitarrini et al., 2014). Naringin (flavanone-7-O-glycoside), hesperidin (3′,5,7-trihydroxy 4′-methoxy flavanones 7-rutinoside), and some plant glucosides were characterized for their capacity to restrain mycotoxin production (e.g., patulin by *Penicillium expansum*, *A*. *terreus*, and *Byssochlamys fulva;* Salas et al., 2012). Similarly, the growth of *A. parasiticus* and its AFB1 production were repressed by methanolic extracts of *Ephedra major* roots (Bagheri-Gavkosh et al., 2009). The inhibition of the growth and AFB1 production of *A. parasiticus* was attributed to quercetin and *p*-coumaric acid flavonoid compounds. In peanut, some stilbenoids (arachidin-1, arachidin-3, and chiricanine A) caused changes in growth rate, mycelial morphology, and spore germination of *A. flavus* (Sobolev et al., 2018). Moreover, a significant decrease or almost complete suppression of AF production was revealed in *A. parasiticus, A. flavus* and *A. nomius* (Sobolev et al., 2018). Similarly, plants with high concentrations of other antioxidants like *β*-carotene, *β*-cryptoxanthin, and total provitamin A also had a reduced amount of AF contamination than hybrids with low carotenoid contents (Suwarno et al., 2019). The relative ease of plant breeding for increased provitamin A as compared to breeding directly for AF resistance suggested novel approaches to suppress AF contamination.

### Masked Mycotoxins

Plants metabolize xenobiotic compounds such as mycotoxins as part of their defense mechanisms. In plants, similar to animals, phase I metabolism (enzymatic transformation such as oxidation, reduction, or hydrolysis), phase II process (sulfatation, glucosidation, glucuronidation) (Coleman et al., 1997; Berthiller et al., 2009), and phase III detoxification (sequestration of compounds conjugated to glucose or glutathione into a vacuole or their permanent attachment to the plant cell wall) (Berthiller et al., 2013) can be differentiated. The chemical transformations in phase I are typical for lipophilic compounds, and most of the hydrophilic compounds are not affected by this phase. In phase I, oxidations are catalyzed by the cytochrome P-450 system, while the hydrolysis is catalyzed by esterases and amidases (Coleman et al., 1997).

Plant-metabolized mycotoxins have been identified mostly for *Fusarium* toxins (HT-2 toxin, T-2 toxin, nivalenol, fusarenon-X, deoxynivalenol, zearalenone, fusaric acid; Berthiller et al., 2013) or insecticidal destruxins from *Metarhizium anisopliae* (Pal et al., 2007). The metabolism of some *Alternaria* toxin derivatives and *Aspergillus* mycotoxins was studied using plant cell cultures (Ruhland et al., 1996) and germinating cereals and vegetables (Ruhland et al., 1997). The same OTA derivatives were isolated from all the tested plant species, and the conversion was nearly complete (Berthiller et al., 2013). However, the quantitative distribution strongly depended on the plant species. In addition to ochratoxin *α*, the main derivatives were (4R)- and (4S)-4-hydroxy-ochratoxin A and β-glucosides of both isomers were detected. Ochratoxin *α* is considered as a non-toxic molecule, whereas hydroxy-ochratoxin A is as potent immunosuppressant as OTA (Berthiller et al., 2013).

The lack of current studies on plant-modified and masked *Aspergillus* mycotoxins calls for attention to a considerable gap in the understanding of mycotoxins’ fate and ecological roles, especially in the case of toxins produced by plant pathogens, such as *A. flavus*.

## Interactions of the *Aspergilli* and Their Mycotoxins With Soil Micro- and Macrobiota

The possible interactions of fungi in the genus *Aspergillus* with the micro- and macrobiota of the soil can be very diverse ranging from direct physical contact, through non-contact biochemical/enzymatic interactions (e.g., *via* biotransformation), up to volatile organic compounds (VOCs) exerting their effects without physical contact between competing organisms.

### *Aspergilli* and Their Mycotoxins Versus Soil Microbiome

Actinomycetes (e.g., Verheecke et al., 2014), Lactobacilli (e.g., Romanens et al., 2019), Bifidobacteria (e.g., Ghazvini et al., 2016), and Bacilli (Siahmoshteh et al., 2017) are the best-studied groups from these aspects. Several studies have conducted screening on microbial collections to find potential biocontrol isolates that inhibit mold growth, testing (1) bacteria ranging from endophytes and rhizosphere species (Wang et al., 2013); (2) traditional fermentation products (Ahlberg et al., 2017); (3) various other samples where natural interactions with toxigenic molds are far less plausible, as in halophilic soils (Jafari et al., 2018) or fish intestines (Veras et al., 2016). The effects on toxin production and the underlying mechanisms of growth and toxigenic nature are, similarly to yeasts, less understood and often not attempted to uncover. OTA biodetoxification was reviewed by Chen et al. (2018) in detail. Microbes can affect OTA concentration by degradation or absorption and at gene regulation level. OTA biosynthesis genes (*acpks, acOTApks*, and *acOTAnrps*) and the general SM regulator *veA* of *A. carbonarius* were downregulated upon co-culturing with *Streptomyces* isolates, with a concomitant decrease in OTA production (El Khoury et al., 2017). While *acOTAnrps* and *acOTApks*, along with *laeA*, a general regulator of fungal secondary metabolism, were found to be downregulated by *Lactobacillus plantarum* (Lappa et al., 2018).

Close physical interaction between bacteria and fungi induced otherwise silent biosynthesis genes in *A. nidulans* (Schroeckh et al., 2009). These are from a wide range of gene clusters known as silent or non-expressed ones of merely predicted SMs (Keller et al., 2005). For example, the direct physical interaction between *A. nidulans* and actinomycetes resulted in orsellinic acid and lecanoric acid production *via* chromatin remodeling (Netzker et al., 2015) of the fungal culture (Schroeckh et al., 2009). Intimate interaction was also described for plant root-*Bacillus subtilis*-*A. niger* interactions, where *B. subtilis* attached on the surface of the plant root and onto fungal mycelia. Transcriptomic data revealed that both the fungus and the bacterium modified their metabolism during the interaction. The antifungal and antibacterial defense mechanisms of both *B. subtilis* and *A. niger* were reduced upon attachment of bacteria to the mycelia (Benoit et al., 2015). Furthermore, bacterial-fungal interaction can also affect plants negatively, for example, *Salmonella enterica subsp. enterica* serovar. Typhimurium established biofilm on *A.niger* hyphae, where the bacterial growth was promoted, while the bacterial biofilm protected the fungus in a mutualistic relationship (Balbontín et al., 2014). Regarding the maize plant, the co-colonization has more adverse consequences on plant growth than colonization by either microbe individually.

Mycotoxins in soil are subjects of microbial biotransformation, detoxification, or degradation. A wide variety of microorganisms can biotransform mycotoxins (reviewed by Verheecke et al., 2016). Most studies were conducted with AFB1 due to its high toxicity and carcinogenicity. Several bacteria and fungi, including *Rhizopus* sp. (Cole et al., 1972)*, Hypomyces rosellus* (*Dactylium dendroides*), and *Corynebacterium rubrum* (Mann and Rehm, 1976) convert AFB1 to aflatoxicol (Figure 1) reducing its C-3 keto on the cyclopentanone ring. AFB1 degradation of *Nocardia corynebacteroides* (*Flavobacterium aurantiacum*) was reported first by Ciegler et al. (1966). However, AFB1 was only metabolized partially and mostly adsorbed to *N*. *corynebacteroides* cells (Line and Brackett, 1995).

Bacteria can reduce the amount of AFB1 by forming AFB2 with lower toxicity, and by making other compounds (AFG2, aflatoxicol) undetectable. *Myxococcus fulvus* reduced AFB1 by 80.7% (Guan et al., 2010). Teniola et al. (2005) studied *Rhodococcus erythropolis*, and a remarkable reduction (70%) of AFB1 was observed with cell-free extracts, and an almost total (over 90%) degradation was detected within 4 h. *Nocardia asteroides* was also able to transform AFB1 to another fluorescent product (Arai et al., 1967).

**Figure 2:**
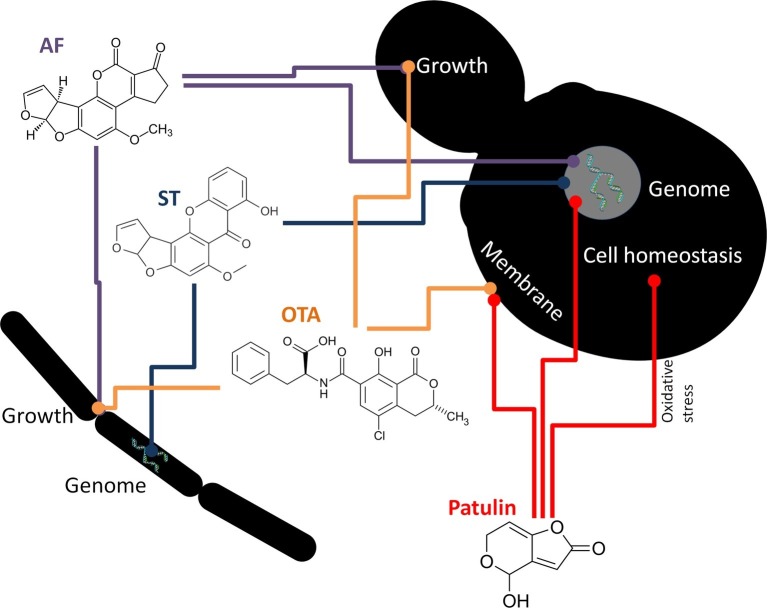
Mechanisms of action of some *Aspergillus* mycotoxins on bacteria (left) and yeasts (right). Colored lines represent antagonistic/damaging effects. AF, aflatoxin; ST, sterigmatocystin; OTA, ochratoxin A.

Among fungi, *Rhizopus* species, such as *R. arrhizus* (Cole et al., 1972), *R. oryzae* (Knol et al., 1990; Faraj et al., 1993; Varga et al., 2005) and *R. oligosporus* (Kusumaningtyas et al., 2006) have been described as being able to degrade AFB1, whereas several other *Rhizopus* species (Cole et al., 1972) also have been shown to remove AFG1. Non-aflatoxigenic *A. flavus* isolates, *Rhizopus* sp., *A. niger*, and *A. glaucus* (*Eurotium herbariorum*) converted AFB1 to aflatoxicol (Figure 1) and vice versa (Nakazato et al., 1990). *Alternaria* sp., *Phoma* sp., *Trichoderma* sp., and *Sporotrichum* sp. have been found to lower AFB1 to 65–99% of the original concentrations (Shantha, 1999). Other fungi, such as *Hypomyces rosellus* (*Dactylium dendroides*) (Detroy and Hesseltine, 1968), *Mucor ambiguous*, *Trichoderma viride* (Mann and Rehm, 1976), *Armillaria tabescens* (Liu et al., 1998), *Phoma* sp. (Shantha, 1999), *Pleurotus ostreatus* (Motomura et al., 2003), and *Trametes versicolor* (Zjalic et al., 2006) have also been described to lower AFB1 concentrations. OTA degradation was demonstrated when applying *Bacillus licheniformis* (Petchkongkaew et al., 2008), *Brevibacterium* species (*B. linens, B. iodinum, B. epidermidis, B. casei*) (Rodriguez et al., 2011), *Acinetobacter calcoaceticus* (Hwang and Draughon, 1994), and *Phenylobacterium immobile* (Wegst and Lingens, 1983). Cell-free supernatants of *Pseudomonas putida* reduced OTA concentration by 8.45–25.70% (Rodriguez et al., 2011). The dimorphic fungus *Apiotrichum mycotoxinivorans* (*Trichosporon mycotoxinivorans*) also degraded OTA (Molnar et al., 2004). *Aspergillus* species such as *A. niger*, *A. fumigatus*, *A. japonicus,* and section *Nigri* species were also able to remove OTA from liquid media (Varga et al., 2000; Abrunhosa et al., 2002, 2014; Bejaoui et al., 2006). Patulin degradation was rarely demonstrated. However, for example, the yeast *Rhodosporidium kratochvilovae* was shown to decrease patulin concentration, whereas the concentration of desoxypatulinic acid increased with time (Castoria et al., 2011). Another possible detoxification mechanism is done by PGUG enzyme from yeast *Meyerozyma guilliermondii* (Chen et al., 2017) or by oxidoreductase from bacteria *Gluconobacter oxydans* (Ricelli et al., 2007). Besides the antagonistic effects of yeasts on mycotoxin production, the cytotoxic and inhibitory effects of the toxins on yeasts (summarized in Figure 2) have also been investigated in some cases (reviewed by Pfliegler et al., 2015). In these studies, the well-known model organisms *Saccharomyces cerevisiae* and the fission yeast *Schizosaccharomyces pombe* have been studied. The toxic effects of AF and OTA, among other mycotoxins, negatively affected the yield of maize mash fermentation processes (Kłosowski et al., 2010), suggesting considerable toxicity. The mechanism of the AF toxic action was shown to be a DNA replication block (Fasullo et al., 2010). Mutagenic effects were detected after ST exposure (Kuczuk et al., 1978). Furthermore, patulin was found to induce oxidative stress and DNA damage both in fission and budding yeasts (Horváth et al., 2012; Papp et al., 2012; Ianiri et al., 2013), with an additional effect of fluidization of the cytoplasm membrane in *S. pombe* (Horváth et al., 2010).

Yeasts utilize general and oxidative stress response pathways along with potential degradation mechanisms to resist mycotoxin exposure (Iwahashi et al., 2006; Ianiri et al., 2013); thus, variation in sensitivity to mycotoxins is not a surprise. Indeed, *Hanseniaspora uvarum*, *S. cerevisiae*, and *Kluyveromyces marxianus* were all found to be resistant to AF and OTA (Angioni et al., 2007). *Aspergillus* mycotoxin toxicity to bacteria is far less understood. Madhyastha et al. (1994) found *Bacillus* and *Brevibacillus* spp. to be highly susceptible to AFB1, but mostly resistant to OTA (except for *B. brevis* and *B. cereus*). Tested strains of *Pseudomonas, Salmonella, Listeria*, and *Escherichia* were usually unaffected by mycotoxins. Additionally, Kuczuk et al. (1978) demonstrated the mutagenic effects of ST on *S.* Typhimurium.

Biodegradation techniques with higher effectiveness may be developed based on existing data and novel research, by further identifying microorganisms capable of biodegrading mycotoxins, by confirming non-toxicity of degradation compounds, by improving both their toxin tolerance and their degradation abilities, and by testing various modes of application.

### Volatile Organic Compounds in Soil Interactions

Fungi interact with plants through VOCs. This phenomenon could play an essential role in fungal pathogenesis. VOCs released by pathogenic fungi could influence plants before any physical interaction between the two organisms. Some VOCs (fatty acid derivatives, terpenoids, phenylpropanoids) are lipophilic; they are small (less than 300 Da) and have high vapor pressure (0.01 kPa or higher at 20°C) and are well known as signal molecules among various organisms. Some of the VOCs (e.g., C_15_H_24_) were found to be unique to aflatoxigenic *A. flavus* (Zeringue et al., 1993). Different fungal-bacterial interaction leads to the specific initiation of fungal SM genes. The two-way volatile interaction between *A. flavus* and *Ralstonia solanacearum*, a similarly widespread and economically crucial soil-borne pathogenic bacterium of peanut, was studied by Spraker et al. (2014). *R. solanacearum* decreased the production of its major virulence factor extracellular polysaccharide in response to *A. flavus* VOCs, while *A. flavus* responded to the bacterial VOCs by reducing conidiospore production and by increasing AF production on peanut. Arbuscular mycorrhizae are also affected by the Aspergilli. *Funneliformis mosseae* (*Glomus mosseae*) decreased the saprobic *A. niger* population through its effect on the plant, whereas *A. niger* inhibited *F. mosseae* in its extramatrical stage through the production of soluble substances or VOCs (McAllister et al., 1995).

**Figure 3:**
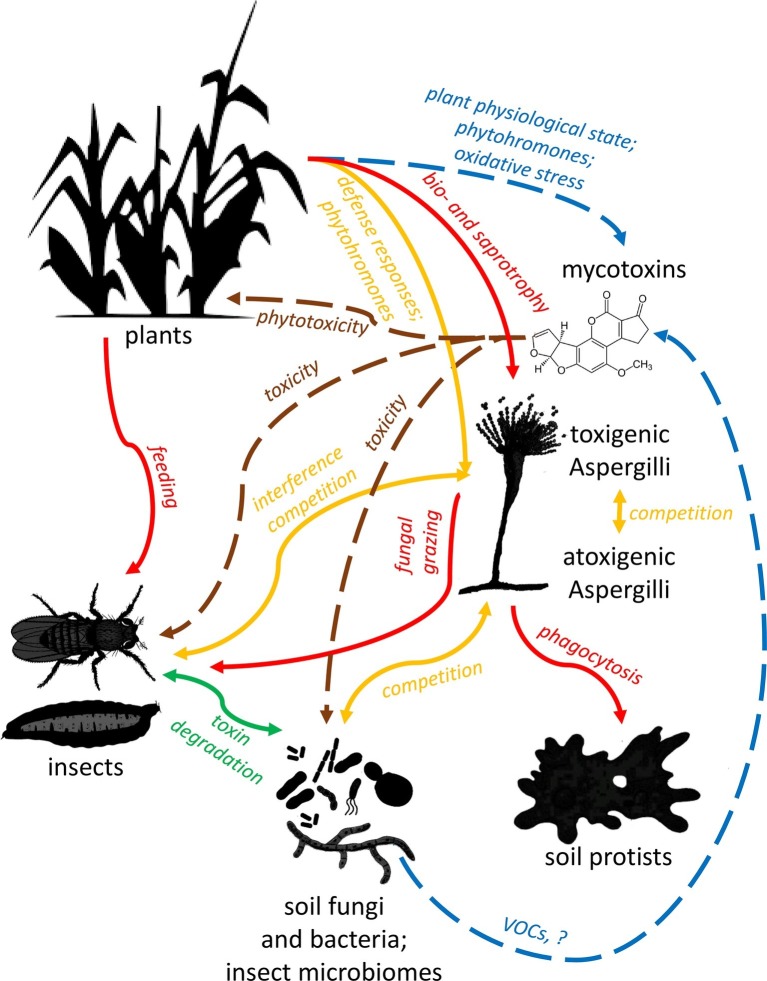
Schematic summary of ecological interactions of plants, fungi, insects, microbes, and *Aspergilli*. Red lines represent trophic relationships, with arrows pointing towards predators and herbivores. Orange lines represent competitive relationships, while green ones show mutualistic relations. Brown lines signal toxic effects of mycotoxins on various organisms, and blue lines show modulating effects of plants and microbes on toxin production. Note that trophic interactions and pathogenicity of soil microbiota are only considered in relation to aflatoxigenic *Aspergilli* and their toxins in this review and figure.

Application of some special yeasts may cause a direct inhibition of mycotoxin production of filamentous fungi, independently of their growth suppressing effect (Petersson et al., 1998; Hua et al., 2014). However, the effect on toxin production is rarely separated from the growth-inhibiting effect due to methodological constraints. *Wickerhamomyces anomalus* (*Pichia anomala*) is the best-characterized yeast species from this aspect. Hua et al. (2014) recognized 2-phenyl ethanol (2-PE), a volatile compound produced by *W. anomalus* as both growth and AF biosynthesis inhibitor in *A. flavus*. AF biosynthesis genes *aflR* (a positive regulator), *aflC* (polyketide synthase, an early gene in the AF pathway), *aflS* (transcription enhancer), *aflK* (versicolorin B synthase), and *aflO* (O-methyltransferase B) were downregulated more than 10,000-fold following 2-PE treatment. Altered expression patterns were also observed for chromatin-modifying genes (MYST1, MYST2, MYST3, *hdaA, gcn5, rpdA*), influencing mold growth negatively (Hua et al., 2014). On the contrary, a subsequent characterization of the temporal transcriptome response of *A. flavus* to smaller, subinhibitory 2-PE concentration revealed inhibition of CPA and AF biosynthesis genes that can be attributed to stimulating active growth of the mold, a condition that does not favor SM production (Chang et al., 2015). These results highlighted the complexity of fungus-fungus interactions depending on the metabolic state and VOC concentration as delicately controlled as the production of mycotoxins (Figure 3).

*Streptomyces* isolates decreased AF levels when co-cultured with *A. flavus*, and this effect was also linked to suppressing AF regulator gene expression (Verheecke et al., 2015). Subsequently, *S. alboflavus* VOCs (mainly dimethyl trisulfide and benzenamine) were shown to play a critical role in this effect, downregulating genes involved in AF biosynthesis in addition to growth inhibition (Yang et al., 2019). Along with *W. anomalus*, *Hanseniaspora uvarum* and *Pichia kluyveri* yeasts were also found to produce VOCs (most notably 2-PE) that hindered the growth and OTA production of *A. ochraceus* (Masoud et al., 2005; Masoud and Kaltoft, 2006). A follow-up study showed that 2-PE inhibition of OTA production byin *A. carbonarius* and *A. ochraceus* isolates was also inhibited by 2-PE, though was caused by the downregulation of their non-ribosomal peptide synthase, polyketide synthase, and monooxygenase genes (Farbo et al., 2018) and the regulatory *veA* and *laeA* genes (Amaike and Keller, 2009).

Another VOC, ethylacetate, was involved in the biocontrol effects of *Saccharomyces*, *Metschnikowia,* and *W. anomalus* yeasts against various molds, including *A. carbonarius* (Oro et al., 2018). VOCs were also responsible for the biocontrol effect of *Candida friedrichii, Candida intermedia, Lachancea thermotolerans*, and *Cyberlindnera jadinii* (Fiori et al., 2014). However, this effect was species-specific. Only *C. friedrichii* reduced mold growth significantly, while the others only inhibited the fungal sporulation.

Finally, it should be noted that yeast-mold, and bacteria-mold interactions through VOCs and other factors, including growth inhibition mechanisms and the mechanisms of gene expression alterations in mycotoxin gene clusters, mostly have been tested in solid and liquid co-cultures, i.e., isolated from the plant host. Studies based on results of the last decades thus should focus on disentangling the interplay among microbes *in vivo*, both to understand the microbial ecology of mycotoxin production in crops and to evaluate the utilization strategies.

### The *Aspergilli* and Their Mycotoxins Versus Protists

Secretion of mycotoxins and escape from phagocytosis are strategies evolved in molds to counter predation in the natural environment. *A. fumigatus* and free-living amoebal species are both abundant soil organisms with antagonistic relationships. Mechanisms of *A. fumigatus* to avoid ingestion by amoebae were modeled with *Acanthamoeba castellanii* (Van Waeyenberghe et al., 2013). Intra-amoebal passage left a fraction of the consumed conidia viable. These spores were able to escape the food vacuoles after phagocytosis and germinated intra-cytoplasmatically, resulting in amoebal death. Interactions with mammalian and avian macrophages and *A. fumigatus* have been compared to these processes, leading to the hypothesis that the ability of the fungus to kill and escape macrophages is a pre-adoptive trait developed in their original ecological niche, namely the soil (Van Waeyenberghe et al., 2013).

Similarly, the slime mold *Dictyostelium discoideum* efficiently consumed fungal spores upon contact with *A. fumigatus,* but the ingestion was more intensive when conidia contained lower amounts of the green spore pigment dihydroxy naphthalene (DHN) melanin (Hillmann et al., 2015). Conidia could survive phagocytosis, and the intracellular germination began only after some hours of co-incubation, which leads to a fatal disruption of the predatory cell. Furthermore, both organisms secreted cross-inhibitory factors that could block fungal growth or induce amoebal aggregation (caused by fungal gliotoxin) with subsequent cell lysis, respectively (Figure 3). *A. fumigatus* and related ascomycetes produced the above mentioned DHN melanin in their spores. However, *A. terreus* is a DHN-melanin synthesis deficient fungus and, instead, had a tyrosinase (TyrP), and an unusual NRPS-like enzyme (MelA) expressed under conidiation. MelA produced aspulvinone E, which is stimulated for polymerization by TyrP. The new pigment, Asp-melanin, in addition to its usual function conferring resistance against UV radiation, hindered phagocytosis by soil amoeba. Contrary to DHN melanin, Asp-melanin did not prevent acidification of phagolysosomes. Therefore, it is probable that it contributes to the endurance of *A. terreus* conidia in an acidic environment (Geib et al., 2016).

Furthermore, the antibiotic compound fumagillin produced by *A. fumigatus* is active against microsporidia and several amoebae but is also poisonous when administered to mammals (Stevanovic et al., 2008). However, this substance was widely used in apiculture against amoebal disease (Bailey, 1955).

### The *Aspergilli* and Their Mycotoxins Versus Arthropods

Recently, roles of fungal SMs in the ecosystem have been demonstrated by toxicological, behavioral, and experimental evolutionary setups with a still limited number of arthropod species. Using fruit fly larvae (*Drosophila*), the role of AF in protection from fungivores is linked to its role in interference competition (Drott et al., 2017), supporting Janzen’s (1977) old and not universally accepted hypothesis (Sherratt et al., 2006). Janzen postulated a fitness advantage of AF production in the presence of soil microbes, vertebrates, or arthropods with which the fungus engages in interference competition. Recent experiments have shown that deterring arthropods indeed confers a fitness advantage to the fungus colonizing nutrient-rich sources (e.g., decaying fruits, seeds, dung, and carrion) (Drott et al., 2017), in addition to the more straightforward and previously described (Caballero Ortiz et al., 2013; Doll et al., 2013) deterring effect on fungal grazers. Mycotoxin production by colonizing fungi may create an adverse environment for arthropods competing for these nutrition sources (Rohlfs and Churchill, 2011). The fact that arthropods, especially insects, are not only competitors of the Aspergilli, but their feeding may predispose the plant or the harvested plant product upon which it feeds to *Aspergillus* infection (Beti et al., 1995; Niu et al., 2008; Ni et al., 2011) further illustrates the complicated tripartite ecological interactions of these molds with plants and arthropods (summarized in Figure 3).

Naturally, the production of AFs may exert selective pressure on exposed arthropods to evolve resistance or tolerance mechanisms that can manifest in detoxification mechanisms or active antagonism towards the fungus. Arthropods are very diverse in their interactions with toxigenic molds, ranging from high susceptibility to remarkable tolerance, presumably, resulting from the variable nature of this evolutionary pressure across habitats. Variation in susceptibility to AF and other mycotoxins has been detected by various studies focusing on mycophagous mites (Racovitza, 2009), *Drosophila* species (Rohlfs and Obmann, 2009), soldier fly larvae (*Hermetia illucens*) (Bosch et al., 2017; Camenzuli et al., 2018), the maize weevil (*Sitophilus zeamais*) (Drott et al., 2017), the yellow and lesser mealworms (*Tenebrio molitor* and *Alphitobius diaperinus*) (Bosch et al., 2017; Camenzuli et al., 2018), the navel orangeworm (*Amyelois transitella*) (Niu et al., 2009), the cabbage looper (*Trichoplusia ni*) (Zeng et al., 2013), or the corn earworm (*Helicoverpa zea*) (Zeng et al., 2006; Niu et al., 2008, 2009). It is plausible that species feeding on highly contaminated food sources are selected towards higher tolerance. Maize weevils are remarkable from this aspect: no mortality increase was observed among these pests even when their food sources contained up to 30,000 μg kg^−1^ AFB1 (Drott et al., 2017).

Additionally, using *Drosophila melanogaster* as a model organism, within-species variation in tolerating mycotoxins has also been observed (Rohlfs, 2006). This intraspecific variation may enable populations to adapt to increased fungal competition and mycotoxin exposure, as demonstrated with the same fly species and *A. nidulans* in an experimental evolutionary setup (Trienens and Rohlfs, 2011). The authors concluded that evolved lineages were more tolerant both to fungal and to purified ST exposure without increased resistance, i.e., without increased ability to impair fungal growth. At the same time, grazing by *D. melanogaster* larvae induced resistance in *A. nidulans*. Grazing activated the expression of many putative resistance genes of the fungus, along with *laeA*, the key SM regulator gene (Amaike and Keller, 2011). The reaction to the fungivores co-occurred with gene expression changes in signal transduction, epigenetic regulation, and SM biosynthesis. Reciprocal insect-fungus interactions may select the Aspergilli for inducible resistance resulting in higher fitness in habitats with a high abundance of fungivores (Caballero Ortiz et al., 2013).

Feeding by *D. melanogaster* larvae induced synthesis of methyl farnesoate and juvenile hormone-III in *A. nidulans* upon expressing a heterologous regulatory protein (Nielsen et al., 2013). It indicates the probable importance of juvenile hormone biosynthesis in fungal-insect antagonistic relationships while also raising possibilities in insecticidal strategies, given the developmental and metabolic importance of juvenile hormones in arthropods (Nielsen et al., 2013). Vice versa, insects may also develop behavioral adaptations to respond to toxic fungal competitors. For example, *Drosophila* larvae have been shown to aggregate around aflatoxigenic *A. nidulans* colonies suppressing fungal growth, improving the chance of larval survival to the adult stage in natural habitats (Rohlfs, 2005; Trienens et al., 2017).

Another fungal-bacterial-insect interaction was described with the connection of an endophytic herbivore, *Dendroctonus rufipennis* (spruce beetle), which is accompanied by an invasion of its galleries by several fungal species (e.g., *A. fumigatus*, *A. nomius*, *Leptographium abietinum*, *Trichoderma harzianum*) (Cardoza et al., 2006). *Trichoderma* and Aspergilli significantly decreased the survival and reproduction of spruce beetle in controlled circumstances. Adult spruce beetle insects exuded an oral secretion, which inhibited the growth of tested fungi except for *A. nomius* or disrupted the fungal morphology in a dose-dependent way. Oral secretions on microbiological media revealed presence of bacteria responsible for the antifungal activity. The isolated bacteria belonged to the Actinobacteria, Firmicutes, Betaproteobacteria, and Gammaproteobacteria taxa that showed species-specific inhibitory activities (Cardoza et al., 2006).

Tolerance requires effective detoxification of food-derived AFs, mechanisms of which have recently been uncovered, but so far only in a few species. *H. zea* has been shown to predispose the plant upon which it feeds to *Aspergillus* infection and concomitant AF contamination, and this pest insect was shown to be able to efficiently metabolize AFB1 into the less toxic AFP1 (Figure 1) using cytochrome P450 monooxygenases (Niu et al., 2008). However, the action of these monooxygenase enzymes is not yet fully understood, as some results indicate that bioactivation, not detoxification may also result from their activity in insects (Zeng et al., 2006, 2013). Larvae of *A. transitella,* a significant pest of almonds and pistachios have been shown to metabolize AFB1 into three biotransformation products, mainly aflatoxicol, and to negligible amounts of AFM1 and AFB2a (Figure 1). The relatively high production of aflatoxicol may reflect a detoxifying adaptation arising from the often mold-infected habitats of the *A. transitella* (Lee and Campbell, 2000). The codling moth *Cydia pomonella*, a pest infecting walnuts and pome fruits, produced none to low levels of AFB1 biotransformation products, suggesting a lower level of detoxification capability (Lee and Campbell, 2000).

A further aspect of insect mycotoxin tolerance and indirect mold-microbiome interactions may also be relevant: the effects of insect symbionts during mycotoxin exposure (Figure 3). Insect microbial symbionts are ubiquitous, incredibly diverse, and their interactions with their hosts are far from being wholly understood (e.g., Dowd and Vega, 2004). At least one symbiotic yeast-like species, *Symbiotaphrina kochii*, can enzymatically detoxify and utilize mycotoxins as carbon sources (along with plant allochemicals and insecticides, even as sole carbon sources) (Shen and Dowd, 1991). More recently, Rohlfs and Kürschner (2010) reported that increased diversity of dietary yeast species benefited *Drosophila* larvae competing with, and exposed to the toxins of *A. nidulans*, by apparently ameliorating the effects of the toxins. These works call attention to the highly under-researched interactions of invertebrate gut microbiotas and toxins. It is plausible that the microbiome of insects and other arthropods, especially of those that are fungal grazers or face interference competition from molds, is an essential factor contributing to the observed variation in resistance to AF and other mycotoxins, and hence the ability of certain arthropods to compete with highly toxigenic molds.

Finally, the application of entomopathogenic fungi is a capable alternative to chemical control of insects, e.g., mosquitoes. *Aspergillus clavatus* from *Oedaleus senegalensis* (Senegalese locust) was highly pathogenic against *Culex quinquefasciatus*, *Aedes aegypti*, and *Anopheles gambiae* mosquito larvae. Application of *A. clavatus* using spore concentrations ranging between 4.3 and 21 × 10^7^ ml^−1^ resulted in 11–68% mortality against *C. quinquefasciatus*, and 37–100% against *A. aegypti* (Seye et al., 2010). Moreover, also in pheromone production, a possible biotechnological application is hiding. The VOC spiroketal (E)-conophthorin (7-methyl-1,6-dioxaspiro[4.5]decane) (Beck and Higbee, 2015) and the isomeric chalcogran are recognized as semiochemicals of some scolytid beetles. Conophthorin is produced by both insects and plants and widely known as a non-host plant VOC from the bark of angiosperm species. Interestingly, VOC production was tested as a response to primary fatty acids of the host plants by non-aflatoxigenic and aflatoxigenic *A. flavus,* as well as *A. niger, A. parasiticus*, *Penicillium glabrum*, and *Rhizopus stolonifera*. On linoleic acid, these fungi formed both spiroketals, while those on linolenic acid emitted only chalcogran. Conversely, no production was detected on palmitic and oleic acid, which also adds a new level of insect-plant-*Aspergillus* VOC interaction (Beck et al., 2012).

Non-aflatoxigenic knockout and low toxin-producing strains of *Aspergillus* are less capable of antagonizing insect populations (Regulin and Kempken, 2018). In addition to balancing selection on mycotoxin production, it must be noted that insect adaptation to mold competition seems to favor tolerance instead of resistance (Trienens and Rohlfs, 2011). Thus, selective pressure on fungi competing with insects is less likely to fuel co-evolutionary arms races or Red Queen dynamics (Rabajante et al., 2015) that would clearly favor more toxigenic strains.

## Conclusions

Because of their economic and public health importance, research on fungal SM mycotoxins has mostly been focused on animal husbandry, the food chain, and human aspects. However, genome data analyses of numerous fungi and the analytical measurements revealed that most of the predicted SM-associated clusters are silent, demonstrating that fungi continue to be a yet undiscovered resource of biologically active molecules. It was also concluded that *A. flavus* might produce metabolites besides well-known mycotoxins that could be underrated contributors to the toxicity to humans and animals. By changing the culture conditions or the genetic regulation to activate silent clusters, new molecules may be discovered that later can be available for medicine or selective biocontrol of fungi or higher eukaryotes.

For a comprehensive understanding of toxigenic molds’ ecology and the evolutionary pressures shaping mycotoxin production, interactions with the micro- and macroflora and fauna in different habitats need to be considered and investigated. The study of the overall role of microbial SMs in natural habitats is now an emerging field. However, the lack of current studies on plant-modified and masked *Aspergillus* mycotoxins calls for attention to a considerable gap in our understanding of mycotoxins’ fate and ecological roles.

Some interaction research revealed new levels of regulations of SM gene expressions through chemical interactions even without direct physical contact. Metabolomic studies at the level of VOCs can boost our knowledge to solve the puzzle of the interactions.

Microbial symbionts of insects are ubiquitous and incredibly diverse; however, their interactions with their hosts are far from being wholly understood. The review also calls attention to the highly under-researched interactions of invertebrate gut microbiotas and mycotoxins. The microbiome of insects and other arthropods is an essential factor contributing to the observed variation in resistance to AF and other mycotoxins, and, hence, in the ability of certain arthropods to compete with highly toxigenic molds.

Recently developed and applied plant protection or soil fertilization agents also should be studied focusing on their effects on interkingdom interactions in soil, or on plants and in plant tissues. In connection with this, the recently approved non-aflatoxigenic *A*. *flavus* strains and fungal preparations are also a subject for further research on interactions of the soil macro- and microbiota. Studying metabolic pathways in pericarp and seeds that are activated differentially by non-aflatoxigenic and aflatoxigenic *A*. *flavus* may help to identify possible target genes to increase plant tolerance and resistance and to fight AF contamination. Mycotoxin biodegradation techniques with higher effectiveness may also be developed based on the existing data and novel research by identifying further microorganisms capable of biodegrading mycotoxins, by improving both their toxin tolerance and their degradation abilities, and by modification of the application.

This article also wanted to attract attention to the fact that most of the direct and indirect yeast-mold and bacteria-mold interactions have been tested only in *in vitro* conditions. Such studies targeted fungal growth inhibition mechanisms and the gene expression alterations in SM gene clusters. Therefore, studies initiated by the results of the last decades should focus on disentangling the interplay *in vivo*, both to understand the microbial ecology of mycotoxin production in crops and to evaluate the utilization strategies. Therefore, greenhouse or microplot experiments should be applied for the extended data collection.

## Author Contributions

IP encouraged TP and WP to investigate the literature on interaction. IP and ZG supervised the writing of this work. TP took the lead in writing the manuscript. WP prepared the figures and wrote sections about yeast-fungal, insect-fungal interactions. TP prepared the sections considering microbial, plant, and soil interactions. ZG prepared the section about masked mycotoxins. All authors discussed the review and contributed to the final manuscript.

### Conflict of Interest

The authors declare that the research was conducted in the absence of any commercial or financial relationships that could be construed as a potential conflict of interest.
